# The Prognostic Role of Angiotensin II Type 1 Receptor Autoantibody in Non-Gravid Hypertension and Pre-eclampsia: A Meta-analysis and Our Studies

**DOI:** 10.1097/MD.0000000000003494

**Published:** 2016-04-29

**Authors:** Jinghui Lei, Yafeng Li, Suli Zhang, Ye Wu, Pengli Wang, Huirong Liu

**Affiliations:** From the Department of Physiology & Pathophysiology (JL, SZ, YW, PW, HL), School of Basic Medical Sciences, Capital Medical University, Beijing, China; Centers for Metabolic Disease Research (YL), Cardiovascular Research, and Thrombosis Research, Department of Pharmacology, Temple University School of Medicine, Philadelphia, PA; Beijing Key Laboratory of Cardiovascular Diseases and Related Metabolic Dysfunction (JL, SZ, YW, PW, HL), Capital Medical University; and Department of Cardiology (HL), Capital Medical University, Beijing, China.

## Abstract

Angiotensin II type 1 receptor autoantibody (AT1-AA) is found in patients with non-gravid hypertension or pre-eclampsia, but the relationship is uncertain.

The aim of the present study was to assess the association between AT1-AA and high blood pressure using meta-analysis, and to evaluate the prognosis value of AT1-AA for hypertensive diseases.

Literature search from PubMed, Embase, and Cochrane databases were conducted using keywords “hypertension” or “pre-eclampsia,” “angiotensin II receptor type 1 autoantibody,” and its aliases from April 1999 to December 2015.

Studies evaluating the association between AT1-AA and non-gravid hypertension or pre-eclampsia were included in this analysis. The quality of the eligible studies was assessed based on the Newcastle–Ottawa Scale with some modifications.

Two researchers then independently reviewed all included studies and extracted all relevant data. Association between AT1-AA and hypertension was tested with pooled odds ratios (ORs) and 95% confidence intervals (CIs). Finally, we evaluated whether AT1-AA predicted the prognosis of hypertension by using a summary receiver-operating characteristic (ROC) curve and sensitivity analysis.

Ten studies were finally included in this meta-analysis. AT1-AA showed more significant association with pre-eclampsia than that with non-gravid hypertension (pooled OR 32.84, 95% CI 17.19–62.74; and pooled OR 4.18, 95% CI 2.20–7.98, respectively). Heterogeneity among studies was also detected probably due to different hypertensive subtypes and AT1-AA measuring methods. Area under summary ROC curve (AUC) of pre-eclampsia was 0.92 (sensitivity 0.76; specificity 0.86). Area under the ROC curve of overall hypertensive diseases or non-gravid hypertension was lower than that of pre-eclampsia (0.86 and 0.72, respectively) with lower sensitivities (0.46 and 0.26, respectively).

The major limitation of this analysis was the publication bias due to lack of unpublished data and the language limitation during literature search. Prospective study with large simple size and specific measuring data collection are needed to enhance our findings in the future.

Our analysis confirms that elevated AT1-AA in serum is significantly associated with hypertensive disorder, especially pre-eclampsia. AT1-AA may be a valuable indicator for poorer prognosis of patients with pre-eclampsia, and could be used in patients with hypertensive disease for risk evaluation and making individual treatment decision.

## Abstract 2

Supplemental Digital Content is available in the text

## INTRODUCTION

Hypertensive disorder is a global concern^1^ and major risk factor for cardiovascular diseases. Long-term hypertension can cause renal arteriosclerosis, subsequent renal insufficiency, and uremia. Distinguished from non-gravid hypertension, pre-eclampsia is defined as high blood pressure and proteinuria during pregnancy, affecting 2% to 8% of pregnancies. It is a leading cause of maternal and fetal high mortality.^2^ So far, the pathogenesis of non-gravid hypertension or pre-eclampsia is not completely clear.

Angiotensin II type 1 receptor (AT_1_R), predominantly expressed in vascular smooth muscle cells, is the central part of renin–angiotensin system (RAS) which plays an important role in blood pressure regulation. The physiological ligand of AT_1_R is angiotensin II (Ang II). Ang II activates a number of cytoplasmic signaling pathways through AT_1_R, including vasoconstriction,^3^ aldosterone synthesis,^4^ and intracellular Ca^2+^ release.^5^ AT_1_R autoantibody (AT1-AA) was firstly discovered by Wallukat in the serum of pre-eclampsia patients.^6^ This autoantibody can bind to the second extracellular loop of AT_1_R and plays an agonist-like effect. As compared with Ang II, AT1-AA has more sustained effect on vasoconstriction^7^ and can cause endothelial cell damage.^8^ These evidences indicate that AT1-AA might contribute to some pathological changes in high blood pressure.

To date, some researchers reported elevated level of AT1-AA in hypertensive patients.^6,9^ However, the exact role of AT1-AA in prognosis prediction of hypertensive disorders is inconsistent. Some of the studies did not show a clear correlation between AT1-AA and high blood pressure. In addition, small sample sizes gave us limitation on any reliable evaluation. Here, by doing meta-analysis, we conducted an assessment for the association between AT1-AA and high blood pressure. Using summary receiver-operating characteristic (sROC) curves, we tested the possibility of AT1-AA as a valuable indicator for poorer prognosis of patients with hypertension.

## METHODS

### Search Strategy

Literature search from PubMed, Embase, and Cochrane databases were conducted using these search terms: “hypertension” or “preeclampsia,” or “pre-eclampsia” or “high blood pressure,” combined with “angiotensin II receptor type 1 autoantibody” or its aliases, such as “angiotensin II type 1 receptor autoantibody” or “autoantibody to the angiotensin II type I receptor” or “AT1-AA” or “AT1 receptor autoantibodies.” Studies between April 1999 and May 2015 were collected, and only language in English and Chinese was chosen.

### Inclusion and Exclusion Criteria

Studies were reviewed by 2 independent researchers. All studies regarding the association between AT1-AA and hypertension or pre-eclampsia were initially included. Inclusion criteria included: standard criteria for non-gravid hypertension (SBP/DBP greater than 140/90 mm Hg) or pre-eclampsia (SBP/DBP ≥140/90 mm Hg and proteinuria after week 20 of pregnancy); reliable AT1-AA measurement with standard criteria for its positive sign. Nonoriginal research (reviews or comments) or animal model studies were excluded. Because AT1-AA was also found in some other diseases such as Graves disease^10^ or Huntington disease,^11^ we also removed studies without matched controls or with hypertensive patients who have complications to avoid misdirection. All studies were subjected to quality assessment, based on the Newcastle–Ottawa Scale (NOS) with some modifications.

### Data Extraction

Data from selected studies were extracted (first author, year of publication, study location and period, subject age, gestational week at sampling for pre-eclampsia study, sex [male %, for hypertension study], sample size, criteria for case/control, AT1-AA measurement and standard criteria for its positive sign, the frequency of AT1-AA positive patients per group).

### Statistical Analysis

An association between AT1-AA and high blood pressure was analyzed with STATA software, version 12.0 (Stata Corp LP, College Station, TX). The pooled odds ratios (ORs) and 95% confidence intervals (CIs) were calculated using the Mantel–Haenszel method. Heterogeneity among studies was estimated with an I^2^ test and a chi-square test. Based on I^2^ values less than or more than 50% and *P* values from the chi-square test that were greater than or less than 0.1, a fixed-effects model or a random-effects model was selected. To eliminate heterogeneity, a subgroup meta-analysis was performed according to disease categories or measurement of AT1-AA. Differences in pooled ORs was estimated using a *Z* test (*P* < 0.05 was considered as statistically significant). A Begg rank correlation test and a funnel plot were used to evaluate potential publication bias.^10^ Sensitivity analysis was used to assess the reliability of the combined results. A sROC curve was also performed using Meta-Disc software.^12^ The area under the curve (AUC) was used to evaluate possibility of AT1-AA for predicting prognosis.

## RESULTS

### Characteristics of Included Studies

Initially, 207 publications were found when we searched from PubMed, Embase, and Cochrane databases using aforementioned strategy. After removal of the duplicate citations, 146 publications remained. Among them, only 39 remained after reviews and comments, and studies not in English or Chinese were excluded by title and abstract screening. Furthermore, 29 of the publications were excluded due to lack of controls, patients with complications, no standard for AT1-AA-positive sign, studies only on mechanism, nonoriginal research, or no specific data. Finally, 10 studies were chosen for the meta-analysis with 757 cases (456 with hypertension only and 301 with pre-eclampsia) and 344 controls. The chart flow of the literature selection was shown in the Guidelines Flow Diagram (see Supplementary Digital Content). Table 1 summarizes the study characteristics. All including studies were assessed in high quality (Table 2).

**TABLE 1:**
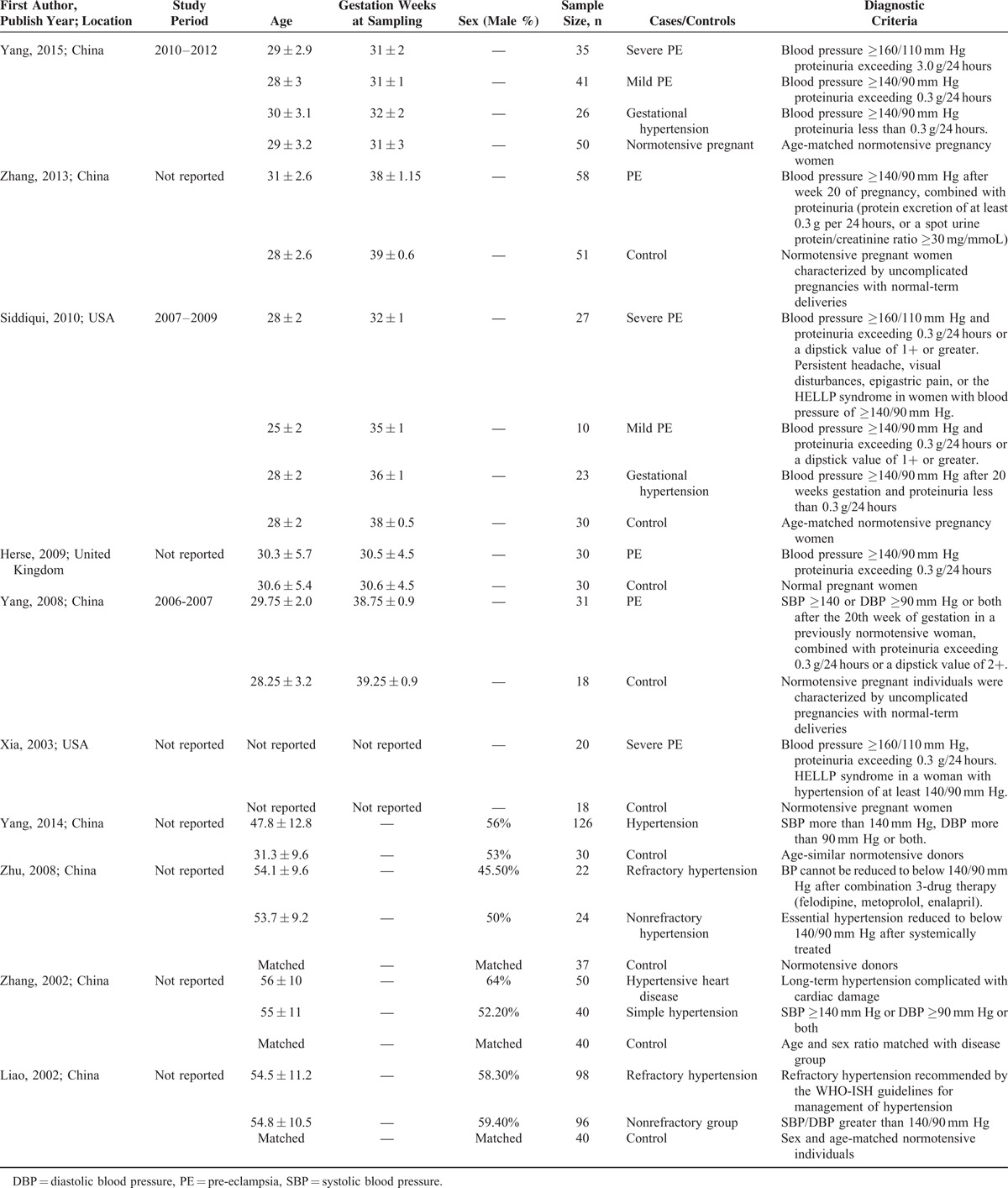
Basic Characteristics of Included Studies

**TABLE 2:**
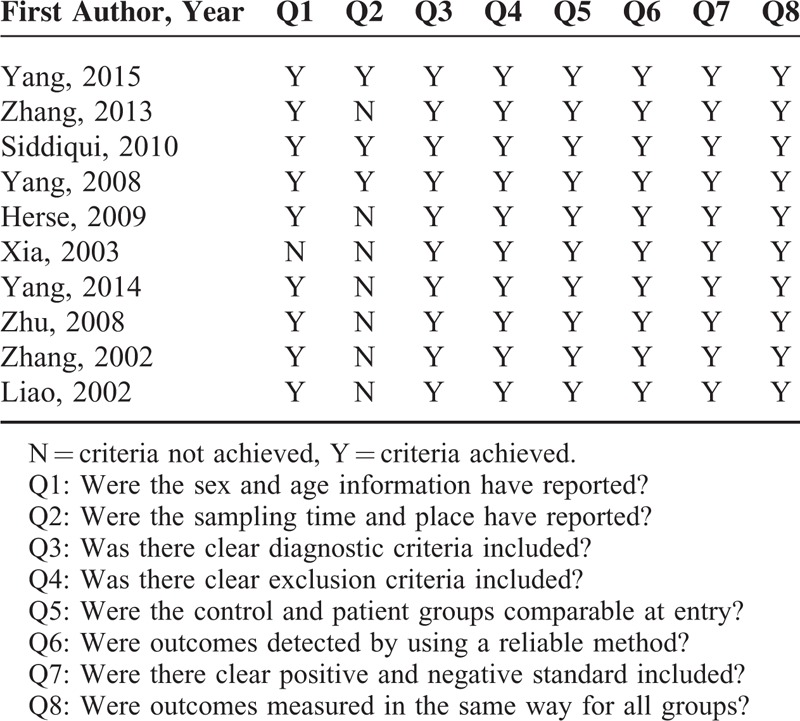
Quality Assessment of Included Studies

### Pooled Analysis for Association Between AT1-AA and High Blood Pressure

**FIGURE 1:**
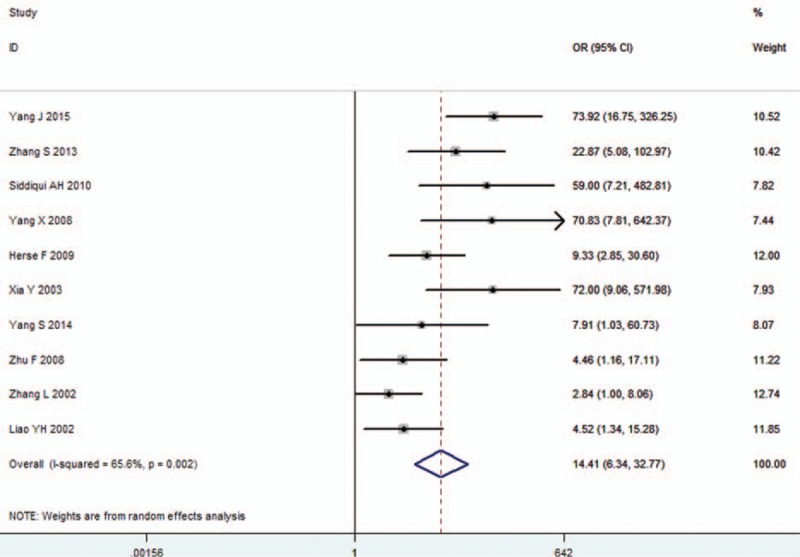
Forest plot of association between AT1-AA and hypertension in all studies. Random-effects model was used due to heterogeneity within each study. AT1-AA = angiotensin II type 1 receptor autoantibody, CI = confidence interval, OR = odds ratio.

A total of 346 (45.7%) in 757 hypertensive patients (including non-gravid hypertension and pre-eclampsia) and 40 (11.6%) in 344 healthy people were AT1-AA-positive. We found the level of AT1-AA was significantly associated with high blood pressure (pooled OR 14.413, 95% CI 6.339–32.771, *Z* = 6.37, *P* = 0.000). Chi-square and I^2^ tests detected slightly heterogeneous among studies (*P* = 0.002, I^2^ = 65.6%); therefore, a random-effect model was chosen (Figure 1).

### Subgroup Meta-analysis for Association of AT1-AA Within Pre-eclampsia or Non-Gravid Hypertension Subgroups

**FIGURE 2:**
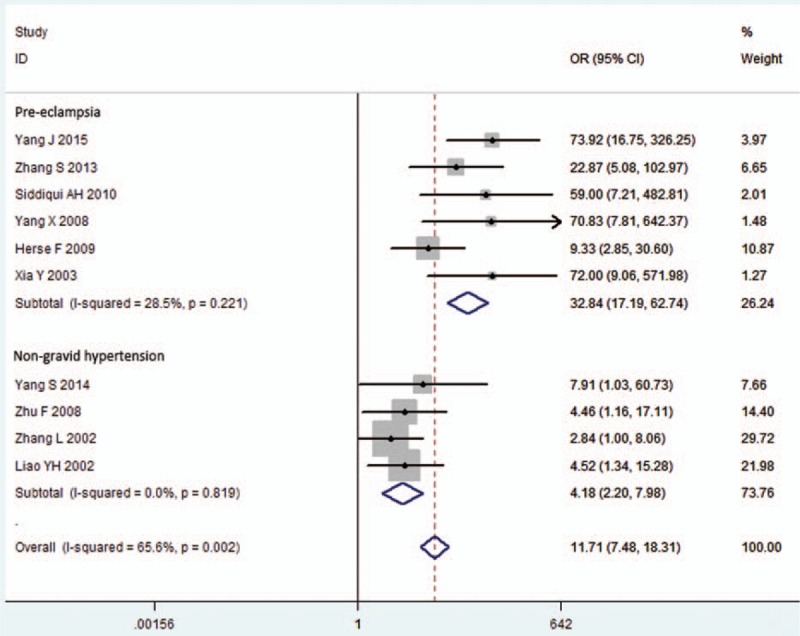
Subgroup meta-analysis for AT1-AA and hypertension. Hypertension was divided into pre-eclampsia and non-gravid hypertension subgroups. Heterogeneity was reduced in each subgroup, so a fixed-effects model was used. AT1-AA = angiotensin II type 1 receptor autoantibody, CI = confidence interval, OR = odds ratio, PE = pre-eclampsia.

As seen in Figure 2, 6 studies were included in the pre-eclampsia subgroup,^13–18^ with 4 in the non-gravid hypertension subgroup.^9,19–21^ A strong association of AT1-AA was found with pre-eclampsia (pooled OR 32.84, 95% CI 17.19–62.74, *Z* = 10.57, *P* = 0.000), but weaker with non-gravid hypertension (pooled OR 4.18, 95% CI 2.20–7.98, *Z* = 4.35, *P* = 0.000). No heterogeneity was found in subgroups (*P* = 0.221, I^2^ = 28.5% pre-eclampsia subgroup; *P* = 0.819, I^2^ = 0.0% non-gravid hypertension subgroup) (Figure 2).

### Meta-analysis for Association Within Different AT1-AA Measurement Subgroup

**FIGURE 3:**
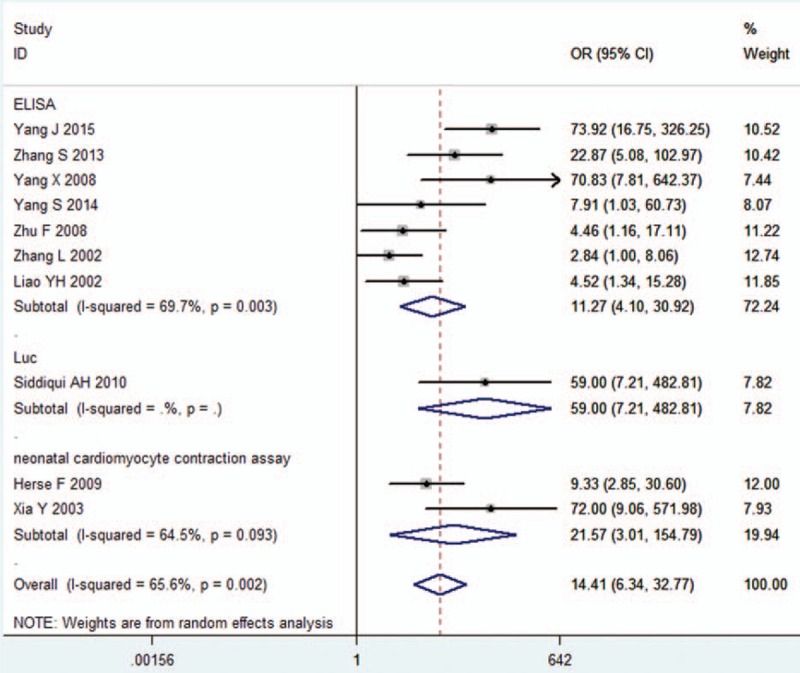
Subgroup meta-analysis by AT1-AA measurement. Ten studies were divided into 3 subgroups, depending on AT1-AA measurements. Heterogeneity was observed in ELISA and neonatal cardiomyocyte contraction assay subgroups. Luc: 4 × NFAT-driven Luciferase reporter assay, AT1-AA = angiotensin II type 1 receptor autoantibody, CI = confidence interval, OR = odds ratio.

Measurement of AT1-AA varied by study: 7 studies used enzyme-linked immunosorbent assay (ELISA),^9,16–21^ 2 used a neonatal cardiomyocyte contraction assay,^13,15^ and only 1 used a 4 × Nuclear factor of activated T-cells (NFAT)-driven Luciferase reporter assay.^14^ As shown in Table 3, the association of AT1-AA with hypertension is independent of AT1-AA measurement. Both ELISA and neonatal cardiomyocyte contraction assay methods detected the association (pooled OR 11.27, 95% CI 4.10–30.92 in ELISA subgroup; pooled OR 21.57, 95% CI 3.01–154.79 in the neonatal cardiomyocyte contraction assay subgroup). However, heterogeneity was observed in each subgroup (*P* = 0.003, I^2^ = 69.7% in ELISA subgroup; *P* = 0.093, I^2^ = 64.5% in neonatal cardiomyocyte contraction assay subgroup) (Figure 3).

**TABLE 3:**
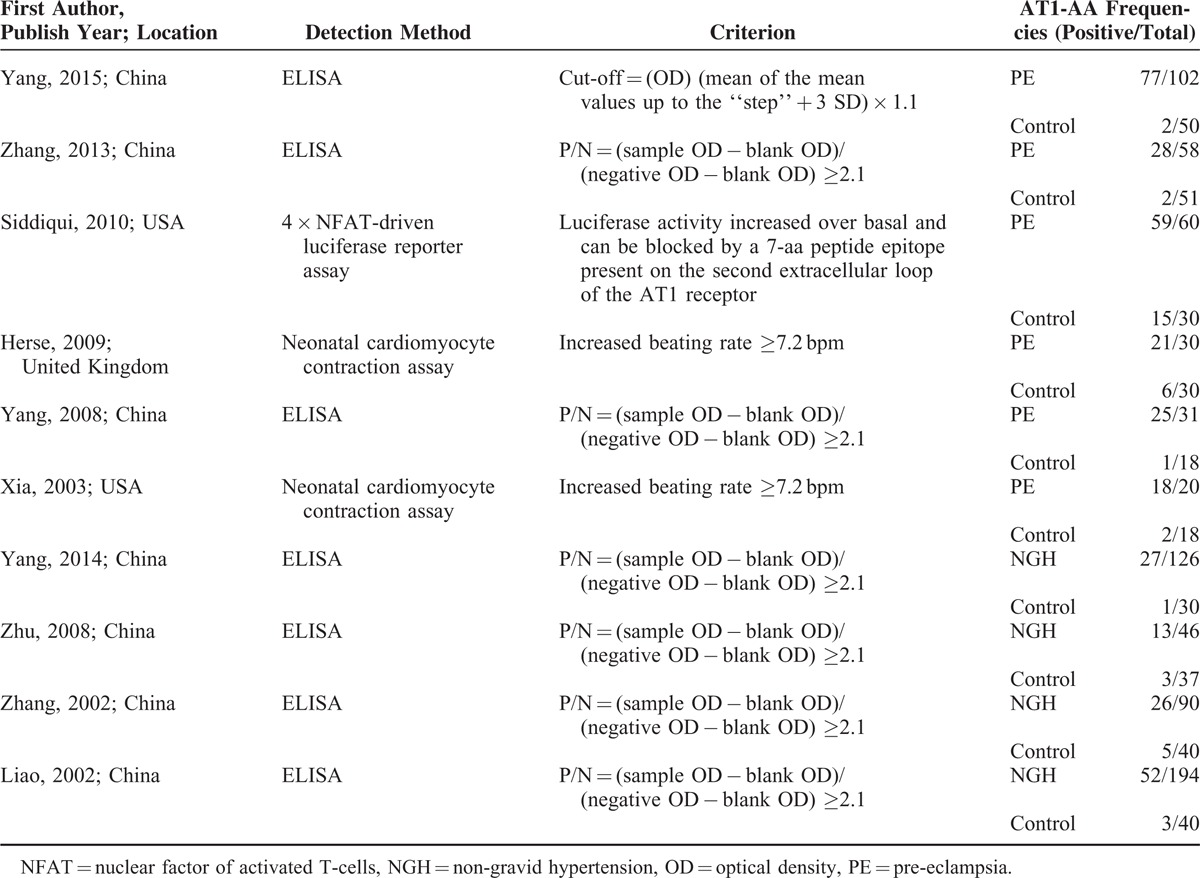
AT1-AA Frequencies in Each Group in Eligible Studies

**FIGURE 4:**
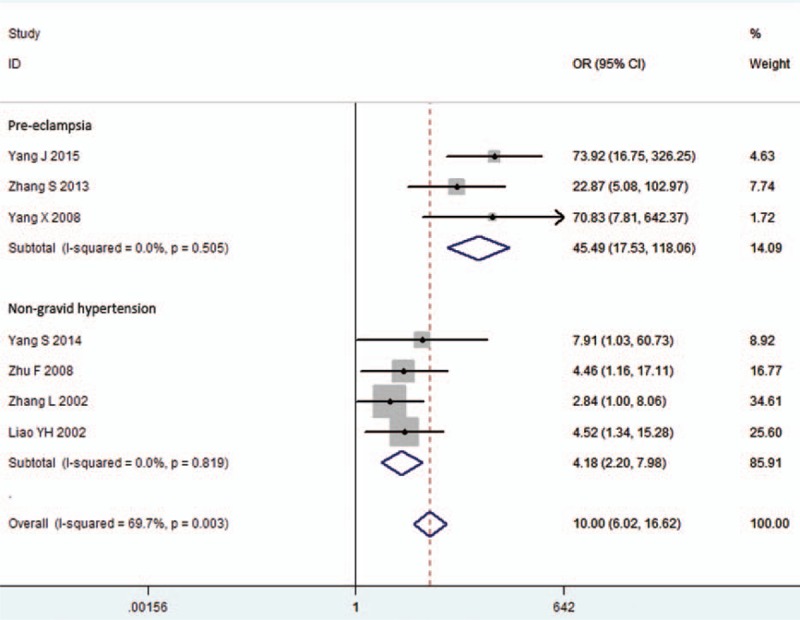
Meta-analysis by pre-eclampsia and non-gravid hypertension subgroup in the ELISA group. Studies included in the ELISA group were divided into 2 subgroups: pre-eclampsia and non-gravid hypertension subgroups. Heterogeneity was not found in both subgroups. CI = confidence interval, ELISA = enzyme-linked immunosorbent assay, OR = odds ratio, PE = pre-eclampsia.

To reduce heterogeneity in ELISA group, a further subgroup meta-analysis according to disease was performed. Significance was found in either pre-eclampsia or non-gravid hypertension group, with pooled ORs as 45.49 (17.53–118.06) and 4.18 (2.20–7.98), respectively. In this subgroup meta-analysis, no more heterogeneity was detected (*P* = 0.505, I^2^ = 0.0% in pre-eclampsia subgroup; *P* = 0.819, I^2^ = 0.0% in non-gravid hypertension subgroup) (Figure 4).

**FIGURE 5:**
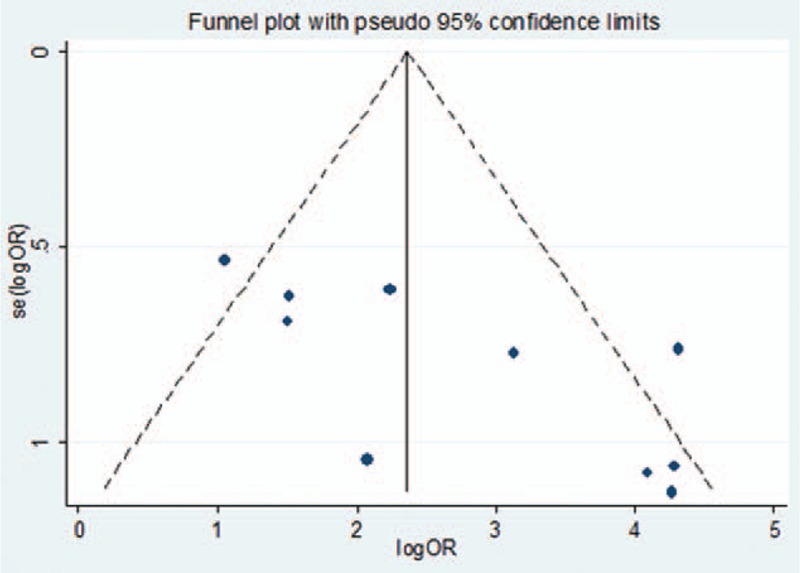
Funnel plot for all studies.

Publication bias among all studies was assessed with a Begg rank correlation test, the result of which was *P* = 0.05. A funnel plot was also used to assess publication bias (Figure 5). Sensitivity analysis showed that the pooled ORs and 95% CIs did not change significantly after any single study was removed (Table 4), suggesting the results were consistent and reliable.

**TABLE 4:**
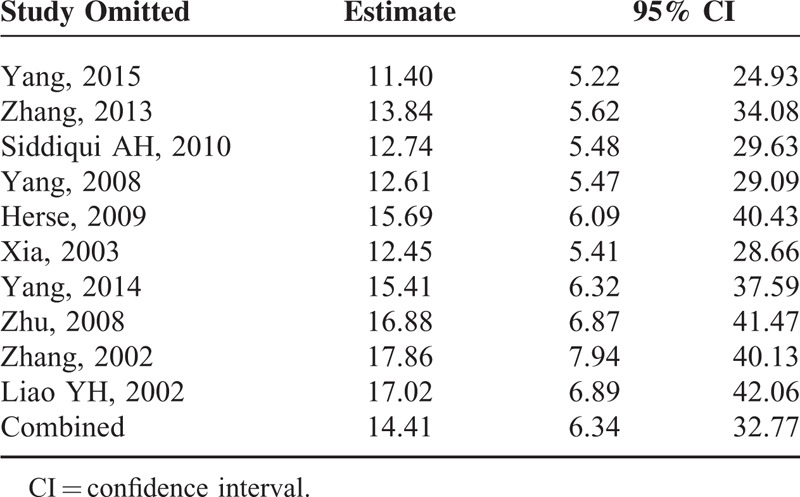
Sensitivity Analysis of Included Studies

### Summary ROC Analysis

**FIGURE 6:**
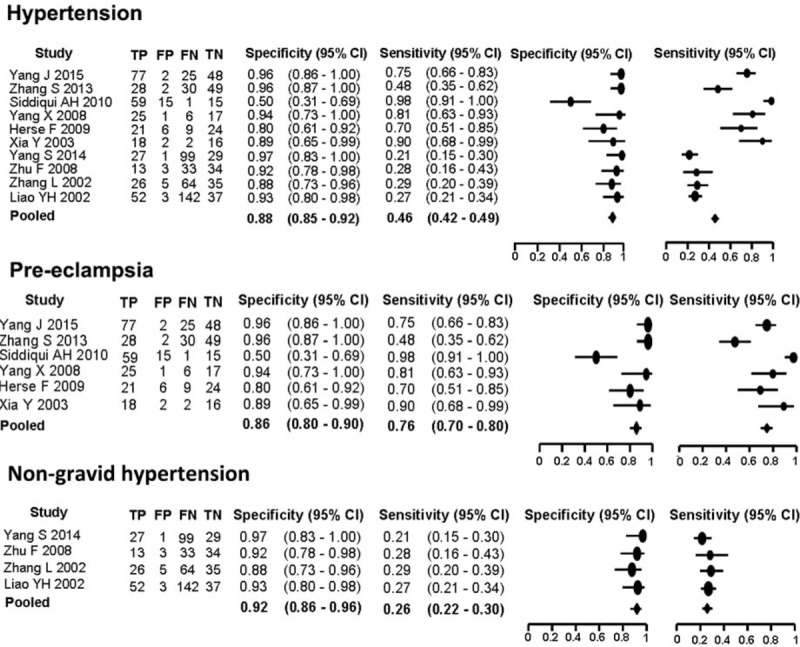
Pooled sensitivity and specificity of AT1-AA in overall hypertension group, or pre-eclampsia subgroup, non-gravid hypertension subgroup. AT1-AA = angiotensin II type 1 receptor autoantibody, FN = false negative, FP = false positive, TN = true negative, TP = true positive.

**FIGURE 7:**
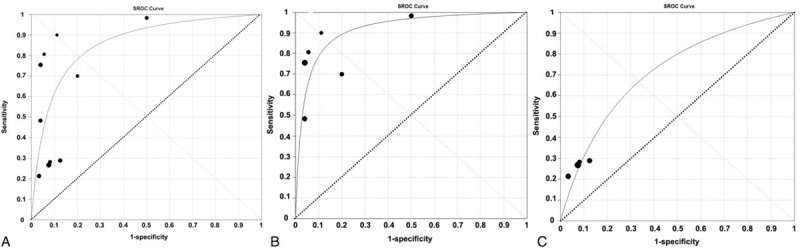
Summary ROC curve for AT1-AA in overall hypertension (A) or pre-eclampsia (B) or non-gravid hypertension (C). AT1-AA = angiotensin II type 1 receptor autoantibody, ROC = receiver-operating characteristic.

Summary ROC analysis combined pooled sensitivity and specificity and was used to assess the possibility of AT1-AA for prognosis prediction. Overall, pooled sensitivity and specificity for AT1-AA were 0.46 (95% CI 0.42–0.49) and 0.88 (95% CI 0.85–0.92), respectively (Figure 6). The AUC was 0.86 (SE 0.04) (Figure 7A).

When the diagnostic performance of AT1-AA for pre-eclampsia or non-gravid hypertension subgroups was calculated independently, pooled sensitivity increased to 0.76 (95% CI 0.70–0.80) for pre-eclampsia subgroup and decreased to 0.26 (0.22–0.30) for non-gravid hypertension subgroup. Pooled specificity slightly changed: 0.86 (95% CI 0.80–0.90) for pre-eclampsia subgroup and 0.92 (95% CI 0.86–0.96) for non-gravid hypertension subgroup (Figure 6). The AUC was 0.92 (SE 0.02) in pre-eclampsia subgroup and 0.72 (SE 0.04) in non-gravid hypertension subgroup (Figure 7B and C).

## DISCUSSION

The present data indicated that AT1-AA is significantly associated with hypertension, especially with pre-eclampsia. A bivariate random-effects analysis strongly suggested that AT1-AA is an indicator for poorer prognosis of patients with pre-eclampsia (summary AUC of 0.92 and a pooled estimate of 0.76 for sensitivity and 0.86 for specificity). Uncontrolled high blood pressure presents a health burden worldwide. Subjects with a history of hypertensive disorders are at increased risk of cardiovascular disease in later life.^22^ Usually, hypertensive patients have to rely on medicaments in their lifetime for blood pressure management. Pre-eclampsia, a pregnancy-specific hypertension that often occurs after 20 weeks of gestation,^23^ seems to be even more intractable due to limited drug options. Therefore, the initial cause of hypertension is an urgent need to be found. Numerous factors were reported to have association with blood pressure regulation, including wide-type of calcium/calmodulin-dependent kinase IV (CaMK4),^24^ platelet antigen 2 (PIA2) polymorphism,^25,26^ and G-protein-coupled receptor kinase 2 (GRK2) overexpression.^27^ These factors were considered to cause vascular impairment through regulating endothelial and vascular smooth muscle function. AT1-AA was detected in the serum of patients with hypertension or pre-eclampsia, and contributed to blood vessel injury. The definite mechanisms of AT1-AA-induced hypertension were hitherto not clear; only several possible pathways have been reported, including vasoconstrictor effect in a sustained manner,^18^ stimulation of vascular smooth muscle cell proliferation and up-regulation of c-fos and c-jun expression,^28^ causing endothelial dysfunction,^8^ increasing intracellular calcium,^29^ stimulating reactive oxygen species (ROS),^30^ and tissue factor expression.^31^ The effect of AT1-AA on aldosterone production also has been reported, but the conclusions are inconsistent. AT1-AA was present in subjects with primary aldosteronism and stimulated aldosterone production,^32^ but in patients with pre-eclampsia, it revealed to decrease aldosterone production.^33^ We have observed both increased and decreased effects of AT1-AA on aldosterone production in our previous study, and reported this effect in a time and dose-dependent manner.^16^ Recently, β-arrestin-1 was reported as a regulator of aldosterone synthesis via G-protein-independent signaling after AT_1_R or β-adrenergic receptor activation.^34,35^ Whether β-arrestin-1 contributes to AT1-AA-mediated aldosterone production through AT_1_R activation needs to be further studied. As AT1-AA can regulate vasoconstriction and aldosterone production, it is tempting to speculate that high level of AT1-AA could play a pathological role in hypertension. To our knowledge, no association study has been done between AT1-AA and hypertension by meta-analysis. The present analysis was designed to assess the clinical significance of AT1-AA in hypertensive disorder. Our data revealed that AT1-AA is significantly associated with hypertension, especially with pre-eclampsia. AT1-AA removal may be a novel therapeutic method for the high blood pressure disorders. In addition to potential risk of AT1-AA in offspring,^36^ we suggested that screening of AT1-AA in pre-eclampsia patients is valuable for their disease prevention and future healthcare.

To address other factors that may affect our results on the relationship between AT1-AA and hypertension pathological features, subgroup analysis was performed. Based on being pregnancy or not, the hypertensive disorders were divided into non-gravid hypertension and pre-eclampsia. Our data revealed that the heterogeneity was observed when meta-analysis was conducted in all studies (I^2^ = 65.6%), but it was eliminated in meta-analyses of each subgroup: pre-eclampsia (I^2^ = 28.5%) and non-gravid hypertension (I^2^ = 0.0%), and an association between AT1-AA and pre-eclampsia (OR 32.84) was much stronger than that between AT1-AA and non-gravid hypertension (OR 4.18). A summary AUC combined with a bivariate random-effects analysis also suggested AT1-AA has prognostic significance for pre-eclampsia, but not non-gravid hypertension. The reason for this state may be because of the differences of subjects between the 2 subgroups: the immune microenvironment in pregnant women is more complex than that in normal people, pre-eclamptic patients were all females aged 20 to 30 years, whereas non-gravid hypertension patients were aged approximately 50 years, and half of them were male. In addition, controls for pre-eclamptic patients were all normal pregnant women, but controls for non-gravid hypertension included both healthy male and female volunteers. A recent research demonstrated the prevalence of maternal transmission in the hypertensive subjects, and highlighted the role of X-chromosome single-nucleotide polymorphisms in this phenomenon.^37^ Interestingly, AT1-AA could be transmitted to offspring from mother via placenta and milk, as was previously reported.^38^ We infer that AT1-AA plays a pathological role in maternal high blood pressure, and also in hypertensive disorders of future generations.

Subgroup analysis was also performed by different AT1-AA measurements: ELISA, neonatal cardiomyocyte contraction assay, and 4 × NFAT-driven luciferase reporter assay. The ELISA method is based on antigen and antibody specificity,^39^ whereas the latter 2 are based on the biological function.^13–15^ Our results suggest that ELISA was efficient for AT1-AA measurement. Because of the simple procedure and repeatable result, we recommended ELISA is suitable for large sample sizes in clinic. The 4 × NFAT-driven luciferase reporter assay and the neonatal cardiomyocyte contraction assay depended on cellular status and experimental environments, so they may be less suitable for large clinical practice, but may be more suitable for mechanism research.

This study has some limitations. First, although the Begg rank correlation test and the sensitivity analysis showed no evidence for publication bias, it is inevitable since we could not include unpublished data. Therefore, the pooled OR may be potentially overestimated. Second, the publication language was limited to English and Chinese; the statistical power of our analysis may be reduced for this reason. Third, studies included in this meta-analysis are retrospective studies, and no prospective study has been published until now; this may reduce the qualities of evidence in clarifying the causal relationship between AT1-AA and high blood pressure. Fourth, the level of AT1-AA was described as “increased” or “positive,” but there was a lack of specific measuring data in these included studies. In this condition, a cut-off value of AT1-AA cannot be established. In addition, the interpretation of different observers or measurement by different methods may influence the results and this is a drawback to clinical applications.

In summary, this meta-analysis including studies revealed that AT1-AA is clearly associated with hypertension, especially pre-eclampsia. With high AUC, high sensitivity, and specificity, we strongly suggest that AT1-AA could be a valuable indicator for poorer prognosis of patients with pre-eclampsia, and could be useful in patients with hypertensive disorders for risk evaluation and making of individual treatment decision.

## Supplementary Material

Supplemental Digital Content
