# Supplementary material for: The Prognostic Role of Angiotensin II Type 1 Receptor Autoantibody in Non-Gravid Hypertension and Pre-eclampsia: A Meta-analysis and Our Studies
Source: Medicine (Baltimore). 2016 Apr 29;95(17):e3494. doi: 10.1097/MD.0000000000003494 (PMC4998714; doi:10.1097/MD.0000000000003494)
Supplement: Supplemental Digital Content [file medi-95-e3494-s001.doc]

Searched by hypertension and aliases: Pubmed: (n=225050)

EMBASE article: (n=96580)

Cochrance library Trial: (n=18236)

Searched by AT1-AA and aliases:

Pubmed: (n=196)

EMBASE article: (n=59)

Cochrance library Trial: (n=5)

Combined with the two search strategy

Pubmed: (n=146)

EMBASE article: (n=58)

Chrochrance library Trial: (n=3)

Duplicate citations removed

(n=61)

Potentially relevant citations identified (n=146)

Excluded based on title and abstract review (n=107)

Review or comment: (n=54)

Non-human studies: (n=49)

Studies not in English or Chinese: (n=4)

Evaluated in full text (n=39)

Excluded after full text review (n=29)

Without control: (n=9)

Without specific data: (n=3)

Not original research: (n=4)

With complication: (n=2)

Mechanism studies: (n=6)

Without judgment standard: (n=5)

Studies included in meta-analysis (n=10)

Figure 1. Flow chart of article search and study selection.
